# A nomogram predicting pneumonia after cardiac surgery: a retrospective modeling study

**DOI:** 10.1186/s13019-024-02797-6

**Published:** 2024-05-31

**Authors:** Kuo Wang, Hai-Tao Zhang, Fu-Dong Fan, Jun Pan, Tuo Pan, Dong-Jin Wang

**Affiliations:** 1 Department of Cardio-Thoracic Surgery, Nanjing Drum Tower Hospital,Affiliated Clinical College of Xuzhou Medical University, Nanjing, 210008 Jiangsu China; 2 Department of Cardio-Thoracic Surgery, Nanjing Drum Tower Hospital, Chinese Academy of Medical Science & Peking Union Medical College, Nanjing, 210008 Jiangsu China; 3 Department of Cardio-Thoracic Surgery, Nanjing Drum Tower Hospital, Affiliated Hospital of Medical School, Nanjing University, Nanjing, 210008 Jiangsu China

**Keywords:** Cardiac surgery, Postoperative pneumonia, Nomogram, Outcomes

## Abstract

**Background:**

Postoperative pneumonia (POP) is the most prevalent of all nosocomial infections in patients who underwent cardiac surgery. The aim of this study was to identify independent risk factors for pneumonia after cardiac surgery, from which we constructed a nomogram for prediction.

**Methods:**

The clinical data of patients admitted to the Department of Cardiothoracic Surgery of Nanjing Drum Tower Hospital from October 2020 to September 2021 who underwent cardiac surgery were retrospectively analyzed, and the patients were divided into two groups according to whether they had POP: POP group (*n*=105) and non-POP group (*n*=1083). Preoperative, intraoperative, and postoperative indicators were collected and analyzed. Logistic regression was used to identify independent risk factors for POP in patients who underwent cardiac surgery. We constructed a nomogram based on these independent risk factors. Model discrimination was assessed via area under the receiver operating characteristic curve (AUC), and calibration was assessed via calibration plot.

**Results:**

A total of 105 events occurred in the 1188 cases. Age (>55 years) (OR: 1.83, *P*=0.0225), preoperative malnutrition (OR: 3.71, *P*<0.0001), diabetes mellitus(OR: 2.33, *P*=0.0036), CPB time (Cardiopulmonary Bypass Time) > 135 min (OR: 2.80, *P*<0.0001), moderate to severe ARDS (Acute Respiratory Distress Syndrome )(OR: 1.79, *P*=0.0148), use of ECMO or IABP or CRRT (ECMO: Extra Corporeal Membrane Oxygenation; IABP: Intra-Aortic Balloon Pump; CRRT: Continuous Renal Replacement Therapy )(OR: 2.60, *P*=0.0057) and MV( Mechanical Ventilation )> 20 hours (OR: 3.11, *P*<0.0001) were independent risk factors for POP. Based on those independent risk factors, we constructed a simple nomogram with an AUC of 0.82. Calibration plots showed good agreement between predicted probabilities and actual probabilities.

**Conclusion:**

We constructed a facile nomogram for predicting pneumonia after cardiac surgery with good discrimination and calibration. The model has excellent clinical applicability and can be used to identify and adjust modifiable risk factors to reduce the incidence of POP as well as patient mortality.

## Introduction

Postoperative pneumonia (POP) is the most prevalent of all nosocomial infections in patients who underwent cardiac surgery [1–3]. Postoperative pneumonia (POP) is the most common infection after cardiac surgery, with a prevalence ranging from 2% to 10%, especially in the first postoperative week [1–7]. Age, smoking history, duration of mechanical ventilation (MV) are well-known risk factors that affect POP. Studies have shown that multiple drug-resistant pathogens can lead to POP, which makes it more difficult to treat, prolongs hospitalization, and increases the mortality rate of patients who develop pneumonia by 5 to 17 times compared with non-pneumonia patients [4–7]. Therefore, many studies have focused on identifying risk factors for pneumonia after cardiac surgery [8–10]. Understanding the risk factors for POP allows for the development of more effective prevention strategies, which are important guides for early identification, targeted prevention and treatment, thereby improving postoperative recovery and quality of life for cardiac surgery patients.

Many risk factors have been reported repeatedly, including advanced age, hypoalbuminemia, hypertension, smoking history, diabetes mellitus, poor cardiac function (NYHA class III-IV), BMI ≥ 24 kg/m^2^, previous cardiac surgery, cardiopulmonary bypass time (CPB time) > 120 minutes, blood transfusion [8–10]. Based on these risk factors, previous studies have constructed some predictive models to assess the risk of pneumonia in patients after cardiac surgery. However, the emergence and prevalence of resistant bacteria significantly increases the risk of POP, and patient characteristics also vary [11]. In addition, with the tremendous advances in surgical and anesthetic techniques, the baseline and comorbidity characteristics of patients have changed greatly in recent years. In addition, malnutrition is prevalent among hospitalized patients with heart failure, significantly increasing the risk of readmission in these patients [12], but few POP-related studies have incorporated this into predictive models. Therefore, there is still a need for an up-to-date study of risk factors for pneumonia after cardiac surgery.

The aim of this study was to identify independent risk factors for pneumonia after cardiac surgery, from which we attempted to construct a nomogram for prediction.

## Methods

### Study design and participants

The data of this study come from the database of patients admitted to the Department of Cardiothoracic Surgery of Nanjing Drum Tower Hospital who underwent cardiac surgery from October 2020 to September 2021 [13]. This study was approved by the Ethical Committee of Nanjing Drum Tower Hospital. All patients included in this study provided written informed consent. Inclusion criteria were patients the ages of 18 and 80 who underwent open-heart surgery, including aortic valve replacement (AVR), mitral valve replacement (MVR), MVR+AVR, aortic surgery + AVR, isolated coronary artery bypass grafting (CABG), valve +CABG surgery, thoracic aortic surgery and others. Exclusion criteria included definite preoperative infection, such as a preoperative temperature of ≥38°C or a white blood cell count above the upper limit of normal, missing perioperative data, and infection due to causes other than pneumonia (eg, isolated surgical site infection and isolated urinary tract infection).

Those who are in violation of medical ethics, have confounding factors that may seriously affect the results, or have poor compliance should be excluded. Those with other research diseases that may have a significant impact on the results of the study or the life and health of the patient. The risk of benefit to patients, such as adverse events, should be weighed. Whether a specific age group can cause bias in trial results. The age range selected for this study was 18-80 years. Natural persons under the age of eighteen are minors, whose cardiorespiratory development is immature and biases the experimental results. In addition, minors can on whether to participate in the experiment, not only to obtain their own consent, but also to obtain their guardian's informed consent and sign the informed consent form, resulting in less enrollment data. Older patients may be in poorer general condition, have more concomitant diseases or co-morbidities, resulting in increased study-related risks and a greater likelihood of adverse events. In addition, the high prevalence of cognitive impairment in the elderly makes it difficult to recruit, to be fully informed, and to make independent decisions about whether to participate in a clinical study, and therefore fewer data are available.

### Data collection

To investigate the risk factors for pneumonia after cardiac surgery, we examined preoperative, intraoperative, and postoperative indicators. Preoperative indicators including age, gender, body mass index, malnutrition, smoking history, hypertension, diabetes mellitus, stroke, myocardial infarction, estimated glomerular filtration rate, sequential organ failure assessment score, left ventricular ejection fraction (%),NYHA class. Intraoperative indicators including emergency surgery, procedure name, minimally invasive, duration of CPB (min), deep hypothermia circulatory arrest, transfusions (%). Postoperative indicators including moderate to severe ARDS, use of ECMO or IABP or CRRT, acute kidney injury, mechanical ventilation time, length of intensive care unit stay (days), death.

### Definition of important variables

We adopted the GLIM criteria as the standard for assessing malnutrition in adult hospitalized patients, and the main element of the GLIM criteria is that the assessment of malnutrition is clearly divided into two steps: "nutritional screening" and "diagnostic assessment". The first step is nutritional screening, which emphasizes the use of clinically validated nutritional screening tools. In the second step, based on a positive screening, the patient is then assessed for malnutrition and graded for its severity. Smoking history referred to previous daily or current smoking. Hypertension referred to previous diagnosis, using antihypertensive medication, or blood pressure ≥140/90 mmHg. Diabetes is diagnosed when a patient has a fasting blood glucose level of 7.0 mmol/L or more, or a blood glucose of 11.1 mmol/L or more two hours after a meal. Stroke is a general term for acute cerebrovascular disease, which is a type of cerebral blood circulation disorders with sudden fainting and unconsciousness, accompanied by crooked mouth, unfavorable speech and hemiplegia as the main symptoms. Myocardial infarction (MI) is defined as ischemic necrosis of the myocardium, which is based on coronary artery disease, in which blood flow to the coronary arteries is drastically reduced or interrupted, resulting in severe and prolonged acute ischemia of the corresponding myocardium, which ultimately leads to ischemic necrosis of the myocardium. The SOFA score, also known as the Sequential Organ Failure Score, is a scoring system for making healing judgments about a patient, which involves determining the degree of impairment of major organ function. The scoring system is divided into six main sections, namely respiratory function, coagulation function, liver function, cardiac system, central nervous system and renal function, with scores ranging from 0-4. The level of the score predicts the mortality rate of septic patients, with the mortality rate increasing by more than 50% for every 30% increase in the score. Other types of cardiac surgery include congenital heart disease (such as ventricular septal defect and atrial septal defect), atrial myxoma, and tricuspid valve replacement, but these procedures are less common in this study. Minimally invasive heart surgery involves a small incision (i.e. an incision) through the chest. In this way, the surgeon is able to reach the heart through the ribs. Blood transfusion is the importation of red blood cells, platelets and cold precipitates. The unit of blood transfusion is % is the volume of blood transfused divided by the total body weight.

Acute Respiratory Distress Syndrome (ARDS) is an acute, diffuse, inflammatory lung injury resulting in increased pulmonary vascular and epithelial permeability, pulmonary edema and gravity-dependent pulmonary atelectasis, and a clinical syndrome marked by intractable hypoxemia. Oxygen levels in the blood can be divided into three categories by comparing the level of oxygen in the blood and the amount of oxygen needed to be given to achieve that level: Mild 200 mmHg < PaO2/FiO2 ≤ 300 mmHg, PEEP or CPAP ≥ 5 cmH2O, and possibly noninvasive ventilation in the mild ARDS group; Moderate: 100 mmHg < PaO2/FiO2 ≤ 200 mmHg, PEEP ≥5cmH2O; Severe: PaO2/FiO2≤100mmHg, PEEP≥5cmH2O. Note: FiO2: inspired oxygen concentration; PaO2: partial pressure of arterial oxygen; PEEP: positive end-expiratory pressure; CPAP: continuous positive airway pressure.

Extracorporeal membrane oxygenation (ECMO) is to draw venous blood out of the body and pump the human oxygenator outside the body, and the oxygenated blood is discharged from carbon dioxide and then infused back into the body, replacing the cardiopulmonary function and gaining valuable time for rescue treatment. Intra-Aortic Balloon Pump (IABP) is one of the left ventricular assist devices, which can improve the balance of oxygen supply and demand of myocardium, increase cardiac output, increase ischemic myocardial perfusion, and promote left ventricular function by increasing coronary blood flow and reducing cardiac afterload. Continuous renal replacement therapy (CRRT) is a treatment that replaces the kidneys to remove metabolites and toxins and correct water, electrolytes, and acid-base imbalances. We put these together considering that these are post-operative invasive procedures that they have a lot in common. The use of these operations can significantly improve the cardiac function status of patients after cardiac surgery, maintain hemodynamic stability, improve systemic tissue perfusion, reduce the amount of vasoactive drugs, and improve the probability of smooth withdrawal and discharge survival of patients. Accurate timing of these procedures, enhanced clinical monitoring, and reduced complications are key to treatment. Acute kidney injury (AKI) was defined as a rise in serum creatinine of ≥26.5 μmol/L within 48 hours or a rise in creatinine to ≥1.5 times the baseline value or a urine output of <0.5 ml/kg-h for 6 hours within 7 days.

### Study outcomes

The primary outcome of the study was pneumonia after cardiac surgery. Pneumonia was diagnosed when the patient meets both clinical and bacteriological strategies. (1): Clinical signs: The presence of a new or progressive radiographic infiltrate plus at least two of three clinical features (fever greater than 38°C, leukocytosis or leukopenia, and purulent secretions) [14, 15]. (2): Pathogenic bacteria were detected twice in sputum culture.

### Statistical analysis

Analyses were performed using R version 4.2.1. Categorical variables were summarized as frequencies (%) and compared using the χ2 or the Fisher’s exact tests, as appropriate. Continuous variables were expressed as median (interquartile range (IQR)) and compared using Mann-Whitney U test. A multivariable logistic regression model was used to assess the independent value of predictor variables. For this analysis, quantitative variables were categorized establishing optimum cutoff thresholds selected from their ROC curves. A backward stepwise approach was followed, including as candidate variables all those that showed univariate significance better than *P*<0.05. Discrimination was measured using the area under the receiver operating characteristic curve (ROC) and its 95% CIs. All reported *P* values are two sides, and values of *P*<0.05 were considered to indicate statistical significance.

## Results

During the study period, a total of 1188 patients who underwent cardiac surgery were included. The incident of POP was 8.8% (105/1188 patients). And mortality rate of patients with POP was 15.2% (16/105 patients), significantly higher than that of patients without POP (OR:17.5, 95%CI (8.0-39.9); *P*<0.001).

The univariate analysis showed that preoperative related factors included age, malnutrition, diabetes mellitus , stroke ,myocardial Infarction (MI), eGFR < 60ml/min, SOFA score ≥ 1 and NYHA class III or IV ; intraoperative correlates included emergency surgery, AVR or MVR, Valve +CABG surgery, thoracic aortic surgery, duration of CPB (min), deep hypothermia circulatory arrest(DHCA) and transfusions (%); and postoperative correlates included moderate to severe ARDS ,Use of ECMO or IABP or CRRT ,acute kidney injury(AKI ),mechanical ventilation (MV) > 48 hours, duration of MV (hours) and length of ICU stay (days), all of which were statistically significant risk factors for postoperative pneumonia. (Table 1).

**Table 1:** Univariate analysis of risk factors of pneumonia after cardiac surgery. Data are n (%) or median (IQR)

	No pneumonia (*n*=1083)	Pneumonia (*n*=105)	*P* value
Preoperative risk factors			
Age (year)	58.00 (50.00, 67.00)	63.00 (56.00, 71.00)	<0.001
Gender (male)	653 (60.3)	68 (64.8)	0.43
BMI (kg/m^2^)	23.95 (21.94, 26.36)	24.03 (21.37, 27.10)	0.64
Malnutrition	72 ( 6.6)	28 (26.7)	<0.001
Smoking history	137 (12.7)	13 (12.4)	1
Hypertension	513 (47.4)	60 (57.1)	0.07
Diabetes mellitus	134 (12.4)	25 (23.8)	0.002
Stroke	78 ( 7.2)	16 (15.2)	0.006
MI	126 (11.6)	30 (28.6)	<0.001
eGFR < 60ml/min	67 ( 6.2)	16 (15.2)	0.001
SOFA score ≥ 1	457 (42.2)	57 (54.3)	0.022
LVEF (%)	55.00 (51.00, 56.00)	55.00 (49.00, 57.00)	0.297
NYHA class III or IV	665 (61.4)	88 (83.8)	<0.001
History of cardiac surgery	68 ( 6.3)	8 ( 7.6)	0.744
Operative risk factors			
Emergency surgery	115 (10.6)	27 (25.7)	<0.001
Procedure name			
Isolated CABG	159 (14.7)	9 (8.6)	0.117
AVR or MVR	416 (38.4)	29 (27.6)	0.038
AVR + MVR	131 (12.1)	6 (5.7)	0.073
Valve +CABG surgery	56 (5.2)	17 (16.2)	<0.001
Thoracic aortic surgery	239 (22.1)	36 (34.3)	0.007
Others	82 (7.6)	8 (7.6)	1
Minimally invasive	194 (17.9)	10 (9.5)	0.041
Duration of CPB (min)	123.00 (85.50, 166.00)	165.00 (126.00, 209.00)	<0.001
DHCA	204 (18.8)	35 (33.3)	0.001
Transfusions (%)	0.50 (0.00, 1.70)	1.50 (0.50, 2.80)	<0.001
Postoperative risk factors			
Moderate to severe ARDS	337 (31.1)	62 (59.0)	<0.001
Use of ECMO or IABP or CRRT	32 ( 3.0)	23 (21.9)	<0.001
AKI	80 (7.4)	22 (21.0)	<0.001
MV > 48 hours	52 (4.8)	36 (34.3)	<0.001
Duration of MV (hours)	8.00 (5.00, 16.00)	19.00 (9.50, 95.00)	<0.001
Length of ICU stay (days)	2.00 (2.00, 3.00)	5.00 (3.00, 13.00)	<0.001
Death	11 (1.0)	16 (15.2)	<0.001

*BMI* Body Mass Index, *MI* Myocardial Infarction, *eGFR* estimated Glomerular Filtration Rate, *SOFA* Sequential Organ Failure Assessment, *LVEF* Left Ventricular Ejection Fraction, *NYHA* The New York Heart Association Functional Classification, *CABG* Coronary Artery Bypass Graft, *AVR* Aortic Valve Repair or Replacement, *MVR* Mitral Valve Repair or Replacement, *CPB* CardioPulmonary Bypass, *DHCA* Deep Hypothermia Circulatory Arrest, *ARDS* Acute Respiratory Distress Syndrome, *ECMO* ExtraCorporeal Membrane Oxygenation, *IABP* Intra-Aortic Balloon Pump, *CRRT* Continuous Renal Replacement Therapy, *AKI* Acute kidney injury, *MV* Mechanical Ventilation, *ICU* Intensive Care Unit

Multivariate analysis of risk factors for pneumonia after cardiac surgery was showed in Table 2, age (>55 years) (OR: 1.83, *P*=0.0225), preoperative malnutrition (OR: 3.71, *P*<0.0001), diabetes mellitus (OR: 2.33, *P*=0.0036), CPB > 135 min (OR: 2.80, *P*<0.0001), moderate to severe ARDS (OR: 1.79, *P*=0.0148), use of ECMO or IABP or CRRT (OR: 2.60, *P*=0.0057) and MV > 20 hours (OR: 3.11, *P*<0.0001) were independent risk factors for POP.

**Table 2:** Multivariate analysis of risk factors for pneumonia after cardiac surgery

	Coefficients ()	Exp ()	95% CI	*P* value	Standard error
Intercept	-4.3427	-	-	-	0.3133
Age > 55 years	0.6024	1.83	1.10-3.12	0.0225	0.2640
Malnutrition	1.3108	3.71	2.10-6.43	<0.0001	0.2553
Diabetes mellitus	0.8455	2.33	1.30-4.08	0.0036	0.2908
CPB > 135 min	1.0309	2.80	1.71-4.68	<0.0001	0.2559
Moderate to severe ARDS	0.5804	1.79	1.12-2.85	0.0148	0.2382
Use of ECMO or IABP or CRRT	0.9572	2.60	1.31-5.10	0.0057	0.3459
MV > 20 hours	1.1348	3.11	1.88-5.12	<0.0001	0.2553

*CPB* CardioPulmonary Bypass, *ARDS* Acute Respiratory Distress Syndrome, *ECMO* ExtraCorporeal Membrane Oxygenation, *IABP* Intra-Aortic Balloon Pump, *CRRT* Continuous Renal Replacement Therapy, *MV* Mechanical Ventilation

**Fig. 1:**
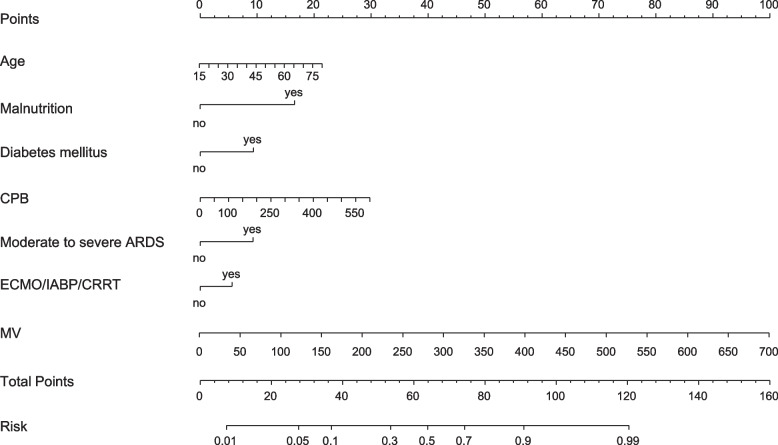
Nomogram based on independent risk factors

**Fig. 2:**
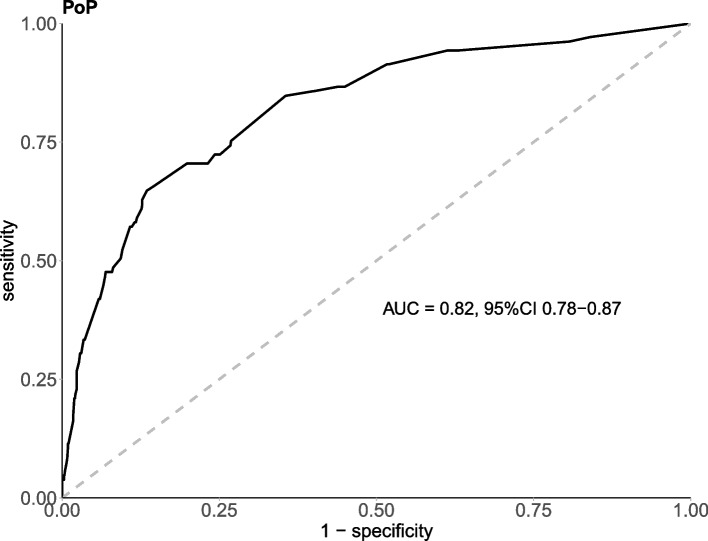
AUC of the nomogram

**Fig. 3:**
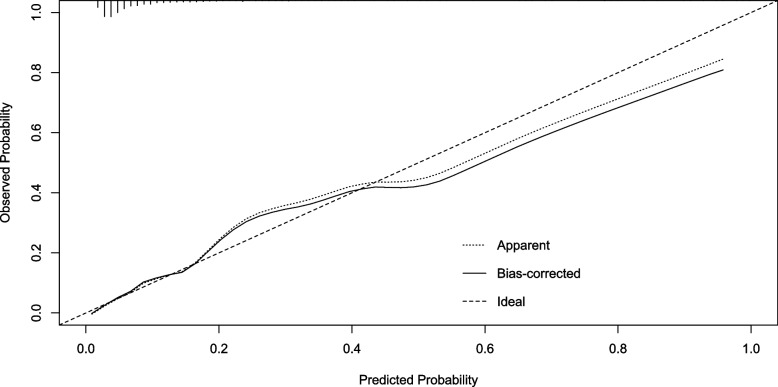
Calibration plot of the nomogram

Based on a multivariate logistic regression model, a nomogram model was developed to predict the probability of POP after cardiac surgery (Fig. 1). The coefficients of these variables were adjusted to a range of scores from 0 to 100, reflecting their relative importance. By summing the corresponding scores, the individualized probability of POP after cardiac surgery can be directly and easily predicted. The nomogram demonstrated excellent discriminate power, with an AUC of 0.82(95% CI 0.78–0.87) (Fig. 2), which confirms the good utility of the model in predicting the development of POP after cardiac surgery. To examine the goodness-of-fit of the model, the calibration plot was used. Calibration plots showed good agreement between predicted probabilities and actual probabilities (Fig. 3).

**Fig. 4:**
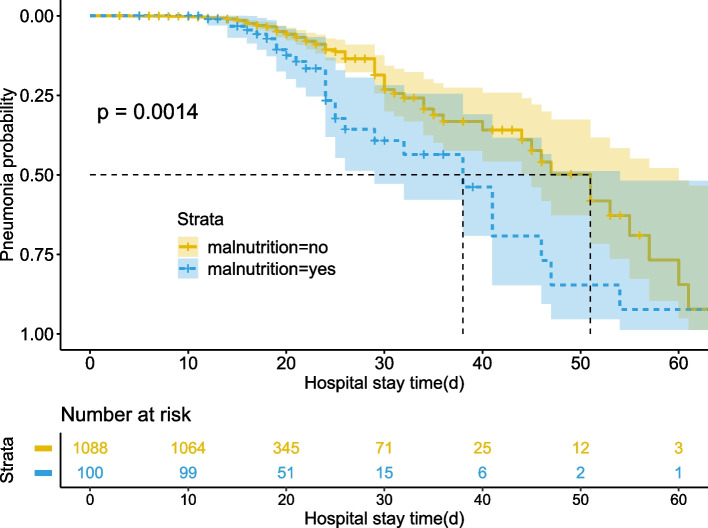
The curve of the incidence of pneumonia with prolonged hospitalization in patients with malnutrition or not

Of interest to us, we found that malnutrition was one of the most important risk factors for POP. We divided the patients into two groups according to whether they were malnourished before surgery. We found that the incidence of pneumonia was significantly higher in malnutrition patients than in patients without malnutrition (Fig. 4).

## Discussion

POP is now considered to be a significant cause of morbidity and mortality in cardiac surgery patients, and this study further confirms this. In our patient cohort, more than half of the infections occurred within 48 hours after the procedure. Infected patients had a significantly higher mortality rate (1.9% vs. 14% P<0.001) and prolonged hospital stay. Significant increases in mortality and other poor prognosis in patients with POP, consistent with the results in the literature, underscore the importance of identifying predictors and high-risk patients [7–9]. Therefore, it is important to diagnose POP as early as possible.

In this cohort study, we evaluated the value of preoperative, intraoperative, and postoperative indicators for predicting POP. We identified that age, malnutrition, diabetes mellitus, CPB, moderate to severe ARDS, use of ECMO or IABP or CRRT and MV time were independent risk factors for POP. Finally, we constructed a simple nomogram and achieved good discrimination and calibration.

This work was based on numerous efforts to optimize perioperative management in cardiac surgery.

A single-center study including 6,222 patients concluded that advanced age, chronic lung disease, peripheral arterial disease, and CPB time >100 minutes, intraoperative red blood cell infusion, and preoperative and intraoperative application of IABP were risk factors for postoperative pneumonia, and a scoring system was developed accordingly. The AUC of the final training set was 0.72, but this retrospective study did not include biomarkers [10]. Another study using 13,380 patients from four centers constructed a model with 10 preoperative and intraoperative risk factors including history of smoking, diabetes, chronic obstructive pulmonary disease, renal insufficiency, age (years), left ventricular ejection fraction, hypertension, CPB time, RBC infusion and history of cardiac surgery and achieved an AUC of 0.84 [16]. The incidence of pneumonia in that study was 6.6%.

This study also suggested that cardiopulmonary bypass lasting more than 2 h was an important cause of postoperative POP, which may be related to the damage to multiple organs in the body by prolonging the duration of cardiopulmonary bypass. Cardiopulmonary bypass can cause ischemia-reperfusion injury and systemic systemic inflammation of cardiomyocytes, resulting in decreased lung compliance. In addition, blood contact with the duct can also cause inflammation in various parts of the body. Kilic et al. found that patients who received CPB for more than 100 minutes had a 1.7-fold increased risk of various complications and POP after cardiac surgery [10]. Allou et al. also reported a positive correlation between CPB duration and POP after cardiac surgery, and in multivariate analyses, the risk of POP increased significantly with increasing CPB duration [17].

Acute respiratory distress syndrome (ARDS) recombines a family of disorders with consequences of pneumonia, alveolar damage, and pulmonary edema [18, 19]. Regardless of the initial lung injury, patients with ARDS are prone to lung infection [18]. While may be due to bronchial contamination due to traditional factors such as endotracheal intubation and duration of mechanical ventilation (MV), but also due to impaired local (alveolar) and systemic defenses, as well as other specific and nonspecific factors [19]. We also found that use of ECMO or IABP or CRRT was independent risk factor for POP. Castelli et al. report that patients who require IABP support after cardiac surgery are more likely to develop postoperative pneumonia because low cardiac output is harmful to the immune system [20]. Bizzarro et al. reported a high prevalence of nosocomial infections of 21% in patients requiring ECMO support, with lung infections being the most common [21]. In addition, critically ill patients treated with CRRT are at risk of lung infection [22]. Patients requiring IABP, ECMO, and/or CRRT are usually in a state of low cardiac output leading to requirement of such support devices and the harmful effects on the immune system. These increased rates of infection with postoperative pneumonia may be associated with concomitant critical illness, prolonged mechanical support, mechanical ventilation (MV) and ICU stay, and impairment of the immune system that promotes the release of inflammatory mediators [20–22]. Extended mechanical ventilation has been reported in many studies to be strongly associated with an increased risk of POP, which is associated with damage to respiratory defense mechanisms caused by endotracheal intubation [23].

We found that malnutrition was a risk factors affecting POP, however, previous studies have rarely looked at the relationship between malnutrition and POP. Malnutrition is a complex organismal state and is recognized as a prognostic risk factor for cardiovascular disease [24]. A study published by Chermesh et al. demonstrated a high risk of complex postoperative course, prolonged ICU stay, and increased 3-year mortality in a population of malnourished cardiac patients [25]. These factors contribute to postoperative pneumonia. Timely identification of patients with malnutrition or at risk of malnutrition and active and effective nutritional therapy is important for improving the clinical prognosis of cardiac surgery patients.

In addition, multidrug-resistant organisms (MDRO) have become the major bacterial group responsible for pneumonia infections in postoperative cardiac patients due to bacterial mutations and overuse of antimicrobial drugs. A study showed that postoperative pneumonia caused by multidrug-resistant microorganisms was associated with adult patients with renal disease, longer intraoperative extracorporeal circulation time, and postoperative nasogastric tubes, with Gram-negative bacilli being the predominant pathogens in patients with pneumonia, with Acinetobacter baumannii having the highest detection rate [26]. Anti-infective therapy should be actively given to reduce the level of inflammatory factors in the blood, improve the patient's immunity, and reduce the probability of MDRO infection in patients with postoperative pneumonia.

The nomogram models can be used in the clinic for joint diagnosis or prediction of disease risk or prognosis by multiple indicators. In addition, the nomogram models can provide an accurate digital survival or risk probability for each patient, which can assist clinicians in decision-making and reflect the idea of individualized medicine. Previous studies have shown that preoperative respiratory physiotherapy and subglottic secretion drainage significantly reduce the incidence of POP, pulmonary atelectasis, and other complications [27–29]. In addition, a growing number of studies have found that aggressive and systematic preoperative oral care plays an important role in preventing and minimizing the development of POP after cardiovascular surgery, with tooth brushing being one of the most acceptable and common measures [30, 31]. However, it may not be appropriate to apply these measures to all patients without selection, as some measures are time-consuming, expensive, and laborious. Therefore, the premise of nomogram application is that there must be clear clinical problems and model construction, and its performance and limitations need to be understood before being applied to clinical decision-making. Only in this way can nomogram be better applied to the clinic.

### Limitations and future directions

Our study also has some limitations. First, the data for this study were derived from a single-center database, with a single patient source, a small study sample size, and the specific cardiac surgical procedures. Second, we lack external validation of the model, which may limit its generalizability. Third, we established standardized diagnostic criteria for POP before the start of the study, but there may be some degree of variability and subjectivity in clinical diagnosis. For example, although we strictly abide by the principles of sputum culture, but because in the retention process is susceptible to oropharyngeal colonization bacteria contamination, its quality is difficult to ensure, false positive rate, may be poorly in line with the diagnosis of clinical infections, and cannot be 100% guarantee of sputum culture positive. This can lead to overestimation or underestimation of the true incidence of POP. A well-designed prospective study may be needed in the future to obtain more accurate estimates of the incidence of POP. Future studies should focus on improving the generalization and practicability of our results.

## Conclusion

Three preoperative indicators (age, preoperative malnutrition, diabetes mellitus), one intraoperative indicator (CPB > 135 min) and three postoperative indicators (moderate to severe ARDS, use of ECMO or IABP or CRRT, MV> 20 hours) were identified as independent risk factors by multivariate logistic regression analysis. A facile nomogram for predicting pneumonia after cardiac surgery was constructed and well validated. A nomogram performed well in terms of calibration and discrimination, and may have good clinical usefulness. Through individualized risk assessment and identification of high-risk populations, nomograms can help clinicians improve clinical decision-making and help patients make informed decisions.

## Data Availability

The datasets generated and/or analyzed during the current study are not publicly available [some patients did not allow us to publish their medical records] but are available from the corresponding author upon reasonable request.
